# Multifunctional cerium-based nanozymes as moonlighting protein mimics for atherosclerosis diagnosis and therapy†

**DOI:** 10.1039/d5sc01014d

**Published:** 2025-04-16

**Authors:** Gui-Mei Han, Jing-Qi Liu, Zhi-Qi Dai, Wei-Liang Jin, Qi-Liang Cai, De-Ming Kong, Li-Na Zhu

**Affiliations:** a State Key Laboratory of Medicinal Chemical Biology, Tianjin Key Laboratory of Biosensing and Molecular Recognition, Research Center of Analytical Science, College of Chemistry, Nankai University Tianjin 300071 P. R. China kongdem@nankai.edu.cn; b College of Chemistry and Chemical Engineering, Qilu Normal University Jinan 250200 P. R. China; c Department of Chemistry, School of Science, Tianjin University Tianjin 300354 P. R. China; d Department of Urology, The Second Hospital of Tianjin Medical University, Tianjin Institute of Urology Tianjin 300211 P. R. China

## Abstract

Moonlighting proteins are multifunctional proteins widely present in organisms, playing crucial roles in various physiological activities. Drawing inspiration from the moonlight proteins, we developed a cerium (Ce)-based nanozyme CF, featuring multiple enzymatic activities along with robust cargo-loading and transport capabilities. The CF was synthesized through a one-step assembly between Ce^3+^ and a phosphorylated amino acid derivative, achieving high biostability through a simple heat treatment. The nanozyme possesses both superoxide dismutase (SOD) and catalase (CAT) activities, enabling scavenging of reactive oxygen species (ROS) and modulation of inflammation by inhibiting NF-κB pathway activation. Besides its enzymatic activities, CF can also serve as a versatile nanocarrier for various cargoes through one-pot co-assembly. Herein, the CF-based nanoassembly loaded with a near infrared fluorescent dye was demonstrated to work well for the diagnosis of atherosclerotic plaques. The nanoassembly co-assembled with probucol exhibited superior ROS-scavenging and anti-inflammatory effects compared to either CF nanozyme or probucol, attributed to the synergy of the nanozyme and the drug, thus facilitating a highly efficient treatment of atherosclerosis. This work introduces a novel Ce-based nanozyme with multifunctional properties, providing a promising approach to endow nanozymes with moonlighting protein-like characteristics, thereby enhancing their functional capabilities and broadening their application potential in various fields.

## Abstract 2

Fabrication of multifunctional cerium-based nanozymes as moonlighting protein mimics for atherosclerosis diagnosis and therapy.

## Graphical abstract

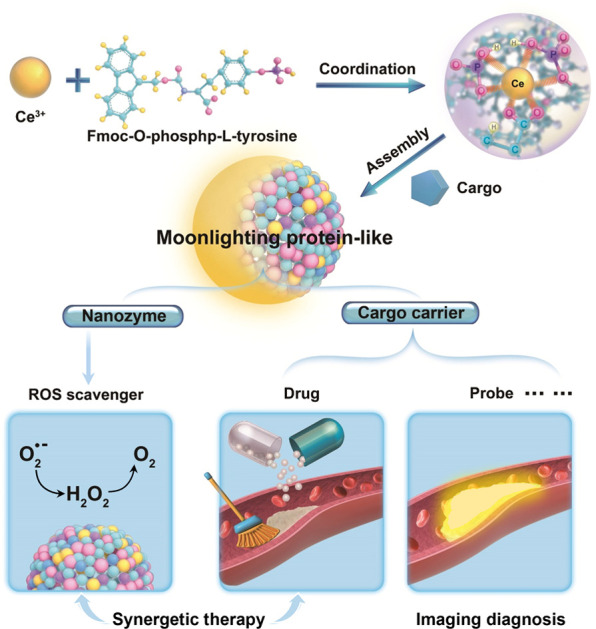

## Introduction

The human body is a complex living organism, containing various types of proteins, each with its own unique function and importance. They not only form the basis of cells and tissues, but also are involved in numerous physiological processes. Besides typical proteins that play a single, well-defined role, these are many multifunctional proteins that can perform two or more distinct functions.^1^ For instance, lactoferrin possesses both deconjugating enzyme and nuclease activities, contributing to the degradation of DNA and RNA, while also functioning as an Fe^3+^ transporter.^2^ ABC transporter proteins not only exhibit ATPase activity but also transport a variety of molecules, including ions, small-molecules, lipids, and drugs.^3^ Phosphoglyceraldehyde dehydrogenase, an enzyme involved in glycolysis, can also act as a transporter protein by transporting tRNA into the nucleus.^4^ These multifunctional proteins, named moonlighting proteins, play crucial roles in many physiological processes, including metabolism, signaling, and cellular stress responses, highlighting the complexity and adaptability of biological systems.^5^ Inspired by this phenomenon in nature, integrating multiple biological functions into nanomaterials to mimic moonlighting proteins is a highly promising direction for future research.

Nanozymes are a class of nanomaterials that exhibit enzyme-like catalytic activities,^6–10^ providing an alternative to natural enzymes and holding significant promise for the development of moonlighting protein-like nanomaterials by integrating additional functions and their enzymatic activities. Some recent reports of multifunctional nanozymes have compellingly demonstrated this potential.^9,10^ Cerium (Ce)-based nanozymes, in particular, can embody multiple enzymatic activities, including oxidase, superoxide dismutase (SOD), and catalase (CAT), because the coexistence of two oxidation states, Ce^3+^ and Ce^4+^, enables reversible redox reactions.^11–16^ This versatility not only renders Ce-based nanozymes highly effective in the treatment of various diseases, but also offers a compelling opportunity to develop moonlighting protein-like nanosystems.^17–20^ However, reports in this area remain scarce, highlighting a significant gap in research.

**Scheme 1:**
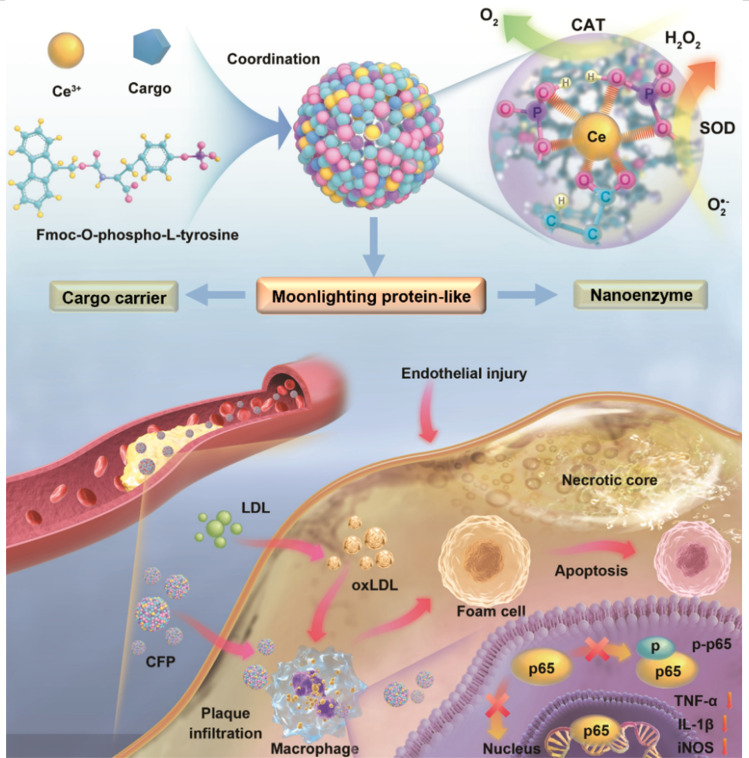
Schematic illustration of the synthesis of the Ce-based nanozyme and the loading of cargoes in the nanozyme through one-pot co-assembly for synergetic therapy of atherosclerosis.

In this work, we aim to incorporate additional functions, such as drug loading and delivery capabilities, into Ce-based nanozymes to create moonlighting protein-mimic nanosystems for the diagnosis and treatment of diseases. To achieve this, a novel Ce-based nanozyme with high biological stability and multiple enzymatic activities was developed through a one-step assembly between Ce^3+^ and a phosphorylated amino acid derivative (Scheme 1), followed by a straightforward heat treatment at 95 °C. The use of the amino acid derivative not only ensures the biocompatibility, but also facilitates the efficient loading of various cargoes into the resultant nanozyme through a simple one-pot co-assembly, thus endowing the nanozyme with dual functionalities: enzymatic activity and the ability to act as a nanocarrier. After a comprehensive study of the nanozyme's enzymatic activity, reactive oxygen species (ROS)-scavenging ability, biostability, and cargo-loading and delivery capacities, we loaded the fluorescent probe IR780 or the clinical drug probucol into the nanozyme, and investigated the feasibility of resultant nano-assemblies for the diagnosis and treatment of atherosclerosis, a disease of the large arteries that is the leading cause of heart disease and stroke. The results demonstrated that the nano-assemblies could efficiently accumulate at the sites of atherosclerotic plaques for imaging diagnosis, and enabled highly effective treatment of atherosclerosis by utilizing the synergy of the nanozyme and the drug.

## Results and discussion

### Construction and characterization of Ce-based nanozymes

**Fig. 1:**
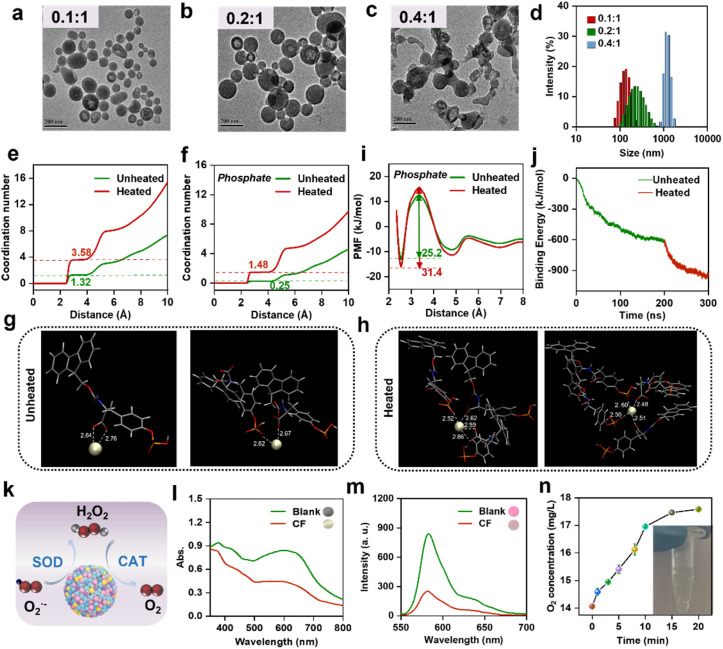
Characterization of the CF nanozyme. (a–c) TEM images of CF prepared using different Ce^3+^ to Fmoc-*O*-phospho-l-tyrosine ratios, (a) 0.1 : 1, (b) 0.2 : 1, and (c) 0.4 : 1. Scale bar: 200 nm. (d) Hydrodynamic diameters of CF prepared using different Ce^3+^ to Fmoc-*O*-phospho-l-tyrosine ratios. (e) Coordination numbers of Ce^3+^ in the CF nanoassemblies with or without heat treatment. (f) Coordination number of Ce^3+^–phosphate in the CF nanoassemblies with or without heat treatment. (g and h) Typical coordination of phosphate and carboxyl around Ce^3+^ in the CF nanoassemblies without (g) or with (h) heat treatment. (i) PMF for Ce^3+^–phosphate. (j) Average binding energies of the CF nanoassemblies with or without heat treatment. (k) Schematic illustration of CF-catalyzed cascade catalytic reactions. (l) SOD activity of CF (inset shows the color photos of reaction solutions). (m) CAT activity of CF (inset shows the photos of reaction solutions). (n) O_2_ generation by CF and H_2_O_2_ (inset shows the photo of O_2_ bubbles generated in reaction solution).

A moonlighting protein-mimic Ce-based nanozyme (referred to as CF) was prepared by the one-step assembly between Ce^3+^ and a phosphorylated amino acid derivative (Fluorenylmethoxycarbonyl (Fmoc)-*O*-phospho-l-tyrosine, Fig. 1a). The use of the amino acid derivative is based on two main considerations: one is to ensure the good biocompatibility of the synthesized nanozyme, and the other is that its aromatic groups can provide cargo-loading sites, which allow cargoes to be easily and efficiently loaded into the CF nanozyme through π–π stacking and hydrophobic interactions, thus endowing the CF nanozyme with moonlighting protein-like multifunctionality.^21,22^ To obtain the CF nanozyme with homogeneous spherical morphology, the feeding ratio of Ce^3+^ to Fmoc-*O*-phospho-l-tyrosine was optimized by keeping the amount of Fmoc-*O*-phospho-l-tyrosine constant. As shown in the transmission electron microscopy (TEM) images (Fig. 1a–c), when the molar ratio of Ce^3+^ to Fmoc-*O*-phospho-l-tyrosine was changed from 0.1 : 1 to 0.2 : 1, the particle size of CF was increased from 76.8 ± 15.8 nm to 117.9 ± 25.5 nm. With the further increase of the feeding ratio to 0.4 : 1, however, large aggregates were observed, accompanied by the formation of precipitation in the reaction solution (Fig. S1†). Correspondingly, the hydrated particle size of CF, measured by dynamic light scattering (DLS), was changed from 134 nm to 235 nm and then to 1.2 µm (Fig. 1d). Since the feeding ratio of 0.1 : 1 gave highly monodispersed CF nanoparticles with the narrowest size distribution, this ratio was used in the subsequent experiments.

Herein, a phosphorylated and Fmoc group-functionalized amino acid derivative was used for CF preparation. In order to elucidate the assembly mechanism of CF, other two amino acid derivatives, Fmoc-l-tyrosine (without the phosphate group) and *O*-phospho-l-tyrosine (without the Fmoc-group), were used to replace Fmoc-*O*-phospho-l-tyrosine (Fig. S2†). By comparing the Tyndall effect of the reaction solutions (Fig. S3†), it was found that neither Fmoc-l-tyrosine nor *O*-phospho-l-tyrosine could efficiently assemble with Ce^3+^, meaning that both phosphate and Fmoc-groups play crucial roles in the assembly of CF nanoparticles, thus indicating that the assembly reaction is synergically driven by the coordination between the phosphate group and Ce^3+^ and the π–π stacking effect of the Fmoc aromatic structure (Fig. S4†). By simulating the dynamic formation of the CF assembly using molecular dynamics (MD) simulations, it was clearly illustrated that Ce^3+^ could coordinate with the O atoms of carboxyl and phosphate groups in Fmoc-*O*-phospho-l-tyrosine. During the formation of the nanoassembly, the Fmoc-*O*-phospho-l-tyrosine molecules started to aggregate and appeared to attach with other aggregates, followed by coordination with surrounding Ce^3+^, leading to the formation of the nanoassembly eventually.

High stability in circulation systems is a prerequisite for nanomaterials as drugs and drug carriers. Due to the crucial role played by Ce^3+^–O coordination during the assembly of CF nanoparticles, the presence of phosphate in the blood circulation system (0.97–1.61 mmol L^−1^)^23^ may adversely affect the integrity of the nanoparticles. By following the escape behavior of fluorescent dye molecules (Fig. S5a†) from the CF nanoparticles, it was found that the CF nanoassembly showed good stability in water, but would be destroyed in PBS buffer, which was clearly reflected by the fluorescence decline due to the release of fluorescent dyes from the nanoassembly (Fig. S5b†). The poor stability in PBS buffer might be attributed to the material disintegration caused by the competition for Ce^3+^ between the buffer's phosphates and Fmoc-*O*-phospho-l-tyrosine molecules. In fact, unsatisfactory biostability is a common problem faced by many coordination-assembled nanomaterials. Fortunately, we found that the biostability of the CF nanoassembly could be significantly improved by an easy way that is, incubation of the nanoassembly at a relatively high temperature for an appropriate time.^24^ As shown in Fig. S6,† after being heated at 95 °C for 45 min, the obtained CF nanoassembly showed excellent stability in different systems, including water, 0.9% NaCl solution, DMEM medium and 2 mM PBS buffer.

Several characterization techniques and MD simulation were used to explore the underlying mechanism. The results of DLS, TEM, X-ray photoelectron spectroscopy (XPS) and X-ray diffraction (XRD) characterization demonstrated that the monodispersity, size, chemical components and crystallinity of CF were barely changed after heat treatment (Fig. S7–S10†). Energy dispersive X-ray spectrometry (EDS) elemental mapping images clearly showed the homogeneous distribution of C, N, O, P and Ce elements in heat-treated CF nanoparticles (Fig. S11†). Having excluded the effect of topology change, we used MD simulation to study the coordination between Ce^3+^ and O atoms of the carboxyl group, as well as between Ce^3+^ and O atoms of the phosphate group in Fmoc-*O*-phospho-l-tyrosine. Fig. S12† displays the radial distribution functions (RDF), represented as *g*(*r*), for the Ce^3+^–carboxyl, Ce^3+^–phosphate, and the total interactions. Based on these RDFs, the coordination numbers of Ce^3+^ were determined. The total coordination number of Ce^3+^ was increased from 1.32 to 3.58 (Fig. 1e), thus signifying a transition to a more stable state as a result of the heat treatment.^25^ Examples of typical coordination of phosphate and carboxyl around Ce^3+^ in the heated or unheated CF are shown in Fig. 1g and h. Notably, the coordination number involving carboxyl groups rose from 1.05 to 2.11 (a 1-fold increase) (Fig. S13†), while the coordination number involving phosphate groups increased from 0.25 to 1.48 (a 4.9-fold increase) (Fig. 1f).

In order to visualize the main source of the increased stability, we calculated the potential of mean force (PMF) for Ce^3+^–carboxyl and Ce^3+^–phosphate. It was found that heat treatment changed the PMF of Ce^3+^–carboxyl from 18.5 to 19.1 kJ mol^−1^ (Fig. S14†). Meanwhile, the PMF of Ce^3+^–phosphate rose from 25.2 to 31.4 kJ mol^−1^ (Fig. 1i). The increased PMF values suggest that the coordination of Ce^3+^ has attained greater energetic stability following the heat treatment.^26,27^ A substantial reduction in the average energy was also observed for the CF nanoassembly after heating, further underscoring the elevated stability (Fig. 1j).

Interestingly, Ce^3+^–phosphate exhibits greater changes in both the coordination number and PMF value than Ce^3+^–carboxyl, suggesting that phosphate contributes more to the improvement of stability during heat treatment. Collectively, Ce^3+^ preferentially coordinates with carboxyl groups during the CF formation at room temperature, and heat treatment contributes more to the coordination between Ce^3+^ and phosphate, which may endow the CF nanoassembly with greater resistance to phosphate-induced disintegration. Overall, a simple heat treatment can greatly promote the coordination of central metal ions, indicating that such treatment may serve as a general strategy to improve the biostability of coordination-assembled nanomaterials.

From the high-resolution Ce 3d XPS spectrum, it could be found that the Ce element in the CF nanoassembly existed in two valence states (Ce^3+^ and Ce^4+^) (Fig. S15†). The reversible interconversion between these two valence states may endow the CF nanoassembly with multiple enzyme activities, such as SOD and CAT activities.^28^ Therefore, CF may function as a multifunctional nanozyme to catalyze cascade chemical reactions (Fig. 1k). The SOD activity and CAT activity could be demonstrated by the nitrogen tetrazolium blue (NBT) photoreduction method and 10-acetyl-3,7-dihydroxyphenoxazine (Amplex™ Red) assay, respectively.^20^ Due to the scavenging of the superoxide anion (O_2_˙^−^), which was produced by the oxidation of riboflavin, by the SOD activity of CF, the blue color of the NBT-O_2_˙^−^ solution faded significantly (Fig. 1l). The CAT activity of CF catalyzed the conversion of H_2_O_2_ to O_2_, thus quenching the fluorescence response of Amplex™ Red to H_2_O_2_ (Fig. 1m), accompanied by the generation of O_2_ bubbles in the reaction system (Fig. 1n). O_2_˙^−^ and H_2_O_2_ are both crucial ROS involved in cellular oxidative damage and inflammation formation. The synergy of SOD and CAT enzymes in organisms can efficiently maintain the redox balance in organisms, which makes CF hold great promise in the treatment of ROS-related diseases, including atherosclerosis.

### CF nanoassembly as a nanocarrier

**Fig. 2:**
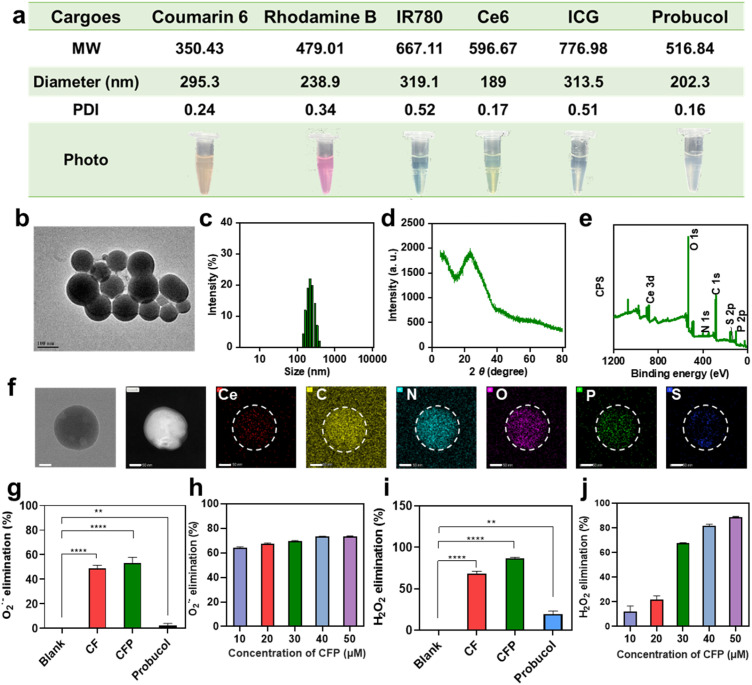
Moonlighting protein-like multi-functions of CF nanoassembly. (a) CF as a nanocarrier for the loading of different cargos (Coumarin 6, Rhodamine B, IR780, Ce6, ICG, and probucol). (b) TEM image of CFP, scale bar: 100 nm. (c) Hydrodynamic diameter of CFP. (d) XRD pattern of CFP. (e) XPS survey spectrum of CFP. (f) EDS elemental mapping images of CFP. Scale bar: 50 nm. (g) O_2_˙^−^-eliminating capabilities of CF, CFP and probucol. (h) CFP concentration-dependent O_2_˙^−^-eliminating capability. CFP concentration is represented as Ce concentration. (i) H_2_O_2_-eliminating capabilities of CF, CFP and probucol. (j) CFP concentration-dependent H_2_O_2_-eliminating capability. CFP concentration is represented as Ce concentration.

The Fmoc-*O*-phospho-l-tyrosine monomer used for the CF assembly contains an aromatic Fmoc structure unit, which can produce strong interactions with many small molecule probes and drugs through synergetic interactions of π–π stacking, hydrophobic interaction and so on, thus realizing the loading of these probes and drugs in the CF nanoassembly.^29^ To verify this, various small probes and drugs, including coumarin 6, rhodamine B, IR780, indocyanine green (ICG), chlorin e6 (Ce6), and probucol, were individually mixed with Ce^3+^ and Fmoc-*O*-phospho-l-tyrosine for the co-assembly reaction. As shown in Fig. 2a, all these small molecule cargoes could be efficiently loaded in the CF nanoassembly through a simple one-pot co-assembly reaction, giving highly monodisperse nanoparticles with enlarged particle size, which was related to the increase of molecular weight and hydrophobicity of the loaded cargoes. These experiments demonstrate that the CF nanozyme holds great potential to work as a general nanocarrier to load different cargoes, thus building different types of moonlighting protein-like multifunctional nanoassemblies. The morphology, monodispersity, and enzymatic activities of CF were well maintained after the co-assembly of different cargoes. As an example, the CFP nanoassembly (P = probucol), which was prepared by co-assembling probucol, a clinical small molecule drug for atherosclerosis treatment, into CF nanoparticles had a regular spherical morphology with a particle size of ∼109 nm (Fig. 2b). It showed a hydrodynamic diameter of ∼202 nm (Fig. 2c) and a relatively homogeneous particle size distribution, with a polymer dispersity index (PDI) of 0.16. Its XRD pattern displayed no obvious crystallization peaks, indicating the amorphous state of the nanoassembly (Fig. 2d). The emergence of the characteristic peak of the S element in the XPS spectrum verified the successful co-assembly of probucol in CFP (Fig. 2e), and EDS elemental mapping images demonstrated the homogeneous distribution of Ce, C, N, O, P and S elements in the nanoassembly (Fig. 2f). High performance liquid chromatography (HPLC) was used to quantify the amount of probucol in the nanoassembly, and the encapsulation rate was calculated to be ∼50.8% (Fig. S16†). The stability of CFP was investigated in 2 mM PBS and 0.9% NaCl solution. After a 24 h incubation, the hydrodynamic diameters of CFP were almost unchanged, accompanied by <5% probucol leakage from CFP (Fig. S17†). It has been reported that atherosclerosis, particularly in its early stages, is accompanied by an increase in phosphate concentration.^30^ To investigate the release behavior of probucol from CFP, we employed the commonly used experimental conditions reported in the literature (10 mM PBS).^31–33^ As shown in Fig. S18,† probucol could be released from CFP at a relatively constant rate in an elevated phosphate environment, achieving an approximately 75% release rate within 24 hours.

The CFP nanoassembly also displayed SOD and CAT activities, and thus ROS-eliminating ability. In addition, due to the presence of probucol, which has been reported to have the ability to eliminate ROS, CFP even showed slightly higher O_2_˙^−^-eliminating ability and significantly higher H_2_O_2_-eliminating ability than CF (Fig. 2g and i), and its O_2_˙^−^ and H_2_O_2_-eliminating efficiencies were both CFP amount-dependent (Fig. 2h and j). When 50 µM CFP, which was represented as Ce concentration, was added, around 70% O_2_˙^−^ and 90% H_2_O_2_ could be eliminated. Since reducing ROS expression is a key step in the treatment of many inflammation-related diseases, the multiple enzymatic activities, together with the drug-loading capability, endow CFP with great promise to work as a moonlighting protein-like nanoassembly for synergistic treatment of these diseases. As an example, its feasibility in the treatment of atherosclerosis, an inflammation-induced disease, will be investigated in this work.

### ROS elimination *in vitro*

**Fig. 3:**
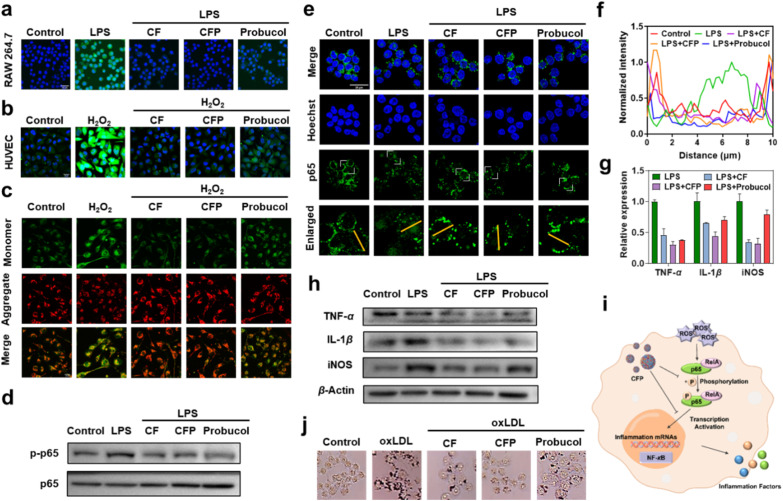
Anti-inflammation by inhibiting the NF-κB pathway. (a and b) ROS-elimination by CF, CFP or probucol from (a) LPS-treated RAW 264.7 cells and (b) H_2_O_2_-treated HUVEC cells. (c) Fluorescence imaging of MMP in HUVEC cells after treatment with H_2_O_2_ or the combination of H_2_O_2_ with CF, CFP or probucol. (d) Western blot images of NF-κB p-p65 and NF-κB p65 in the RAW 264.7 cells after treatment with LPS or the combination of LPS with CF, CFP or probucol. (e) Immunofluorescence assay of nuclear translocation of NF-κB p65 in the RAW 264.7 cells with different treatments. Scale bar: 25 µm. (f) Plot profile of the interested region (yellow line labeled) in the enlarged CLSM images of (e). (g) Inflammatory factor-related gene expression levels in the RAW 264.7 cells with different treatments. (h) Inflammatory factor-related protein expression levels in the RAW 264.7 cells with different treatments. (i) Schematic illustration of the activation of the NF-κB pathway. (j) Inhibition of oxLDL-induced foam cell formation by CF, CFP and probucol. Scale bar: 25 µm.

Atherosclerosis is caused by chronic inflammation in the artery walls.^34–36^ Elevated ROS level is an important marker of atherosclerotic plaque formation and increased plaque vulnerability risk,^37–41^ and antioxidant therapy has become a promising way for the treatment of atherosclerosis.^40,42^ Having demonstrated the superior ROS-eliminating ability of CFP compared to CF in solution, we then compared their ROS-eliminating capabilities in living cells. Human umbilical vein endothelial cells (HUVEC) were treated with H_2_O_2_ to increase intracellular ROS expression by introducing exogenous ROS,^39^ and murine macrophage cells (RAW 264.7) were treated with lipopolysaccharide (LPS) to induce the enhancement of endogenous ROS.^38^ When these cells were further incubated with CF, CFP or probucol, similar results were obtained. That is, CF, CFP and probucol were all able to reduce the intracellular ROS levels in H_2_O_2_ or LPS-treated cells, and the ROS-eliminating ability of CFP was obviously better than those of CF and probucol due to the synergetic effects of the nanozyme and the drug (Fig. 3a, b and S19†).

The above experiments demonstrate that the CFP nanoassembly can effectively remove both exogenous and endogenous ROS from cells, which can not only alleviate ROS-induced cellular damage, but also inhibit the formation of foam cells, thus contributing to the prevention and treatment of atherosclerosis. Elevated ROS levels in cells may cause changes in the mitochondrial membrane potential (MMP) and thus induce cell damage. By using the JC-1 kit to detect the MMP changes, it was found that H_2_O_2_ treatment resulted in the decrease of red fluorescence (aggregates) and the increase of green fluorescence (monomers), indicating ROS-induced MMP depolarization. Such a MMP depolarization could be alleviated by CF, CFP and probucol. And again, CFP gave the best alleviating effect (Fig. 3c).

### Anti-inflammation by inhibiting the NF-κB pathway

Having demonstrated the ROS-eliminating ability of CFP, we next investigated its anti-inflammation function. It has been reported that ROS can regulate the secretion of downstream inflammatory factors and trigger an inflammatory response, thus promoting the development and progression of atherosclerosis by activating the intracellular NF-κB inflammatory pathway.^43,44^ The activation of the NF-κB pathway is typically characterized by the phosphorylation and nuclear translocation of NF-κB p65,^45–47^ a subunit of the NF-κB transcription factor family. Thus, the inhibiting abilities of CFP to NF-κB p65 phosphorylation and nuclear translocation were studied. The western blot test showed that LPS induced the improvement of the NF-κB p65 phosphorylation level in RAW 264.7 cells, which is reflected by the increased phosphorylation product of NF-κB p-p65 (the p-means phosphorylated), but such a phosphorylation process could be efficiently overcome by the CF nanozyme, probucol and their assembly CFP (Fig. 3d and S20†). In the immunofluorescence assay, obvious fluorescence, which was caused by the NF-κB p65 nuclear translocation, was observed in the nucleus of LPS-treated RAW264.7 cells. And again, such a nuclear translocation was significantly inhibited by CF, CFP and probucol, especially CFP (Fig. 3e). The phenomenon could be seen more clearly in the enlarged images. The extracted fluorescence intensity of p65 in the plot profile is also shown in Fig. 3f. Collectively, the highly efficient inhibiting capabilities to NF-κB p65 phosphorylation and nuclear translocation mean that CFP can turn off the NF-κB pathway, thus holding great promise in regulating the secretion of downstream inflammatory factors, such as TNF-α, IL-1β and iNOS. The results of qPCR and the western blot test demonstrated that CFP could indeed down-regulate the expression levels of inflammatory factor-related genes (Fig. 3g) and corresponding proteins (Fig. 3h and S21†), thus holding great promise in anti-inflammatory therapy. Upon a thorough evaluation of the above experimental results, it is evident that CFP demonstrates superior anti-inflammatory effects by inhibiting the NF-κB pathway (Fig. 3i).

It is well known that the development and progression of atherosclerosis is inflammation-related, and atherosclerotic plaques originate from the formation of foam cells.^48^ The excellent anti-inflammatory activity makes CFP a promising candidate drug for atherosclerosis prevention and treatment. To demonstrate this, its inhibiting ability against foam cell formation was investigated. As shown in Fig. 3j and S22,† in oxidized low density lipoprotein (oxLDL)-incubated RAW264.7 cells, a large number of lipid droplets were observed, which is an important marker of foam cell formation.^49^ Treatment with CF or probucol could reduce the accumulation of lipid droplets in cells, thus preventing the formation of foam cells to some degree. Compared to CF and probucol, CFP showed much stronger ability, and the formation of foam cells was nearly completely inhibited, which can also be attributed to the synergy of multiple enzymatic activities and the drug probucol. This experiment confirmed the potential of CFP for the prevention and treatment of atherosclerosis at the cellular level.

### Biocompatibility, intracellular uptake and accumulation in atherosclerotic plaques

**Fig. 4:**
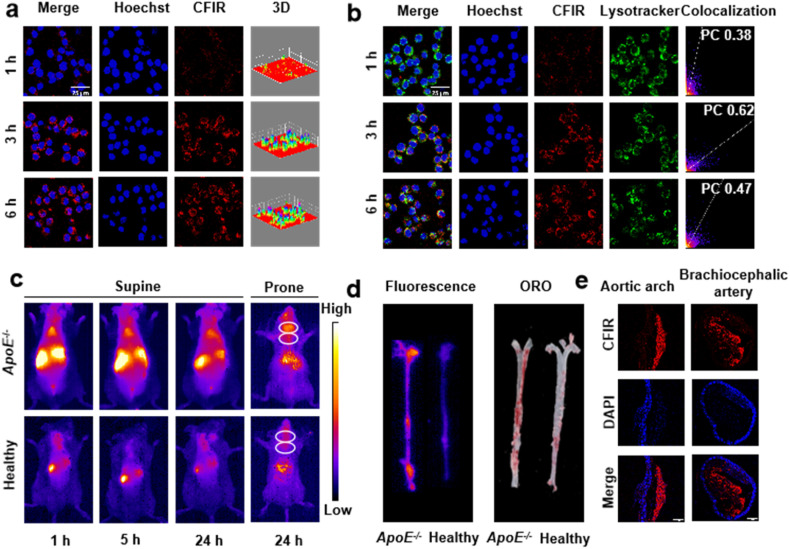
Biocompatibility and intracellular uptake of CFIR and its accumulation in atherosclerotic plaques. (a) CLSM images of RAW 264.7 cells treated with CFIR. (b) Fluorescent colocalization of lysosomes and CFIR. PC values are given in the right images. Scale bar: 25 µm. (c) *In vivo* images of healthy C57BL/6J mice and atherosclerotic *ApoE*^−/−^ mice after tail injection of CFIR. (d) *Ex vivo* fluorescence and ORO images of the aorta obtained after 24 h injection. (e) Tissue section staining of the aorta.

The exciting *in vitro* results encouraged us to validate the *in vivo* potential of CFP for the treatment of atherosclerosis. Before this, the toxicity, biocompatibility and atherosclerotic plaque-targeted ability of the CFP nanoassembly were examined. Cytotoxicity experiments demonstrated that the CFP nanoassembly presented very weak or negligible cytotoxicity (Fig. S23†). Hemolysis experiments showed that CFP had no hemolysis reaction even at the concentration up to 200 µM (Fig. S22†). To visually observe the atherosclerotic plaque-targeted ability of CF-based nanoassemblies, near infrared fluorescent probe IR780 was co-assembled into CF to prepare the CFIR nanoassembly. Similar to CFP, the obtained CFIR also showed the spherical morphology with good monodispersity (Fig. S25†). The results of confocal laser scanning microscopy (CLSM) showed that the CFIR nanoassembly could be taken up by macrophages, and a sufficiently high uptake rate was achieved after incubating the cells with CFIR for 3 hours (Fig. 4a). By following the co-localization of lysosomes with the CFIR nanoassembly (Fig. 4b), it could be found that the Pearson's correlation coefficient (PC) increased from 1 h to 3 h and then declined from 3 h to 6 h (0.38 → 0.62 → 0.47), indicating that CFIR nanoparticles were taken up through lysosomal endocytosis then escaped from lysosomes to the cytoplasm.^47^

The targeted accumulation behavior of CF-based nanoassemblies was also evaluated by *in vivo* experiments. All animal studies were performed according to the guidelines set by the Tianjin Committee of Use and Care of Laboratory Animals, and the overall project protocols were approved by the Institute of Radiation Medicine Chinese Academy of Medical Sciences. The accreditation number of the laboratory is SYXK(Jin) 2019-0002 promulgated by Tianjin Science and Technology Commission. After the successful establishment of atherosclerotic plaques models, CFIR was administered to mice *via* tail vein injection. As shown in Fig. 4c, enhanced IR780 fluorescence was clearly observed in the atherosclerotic plaques of apolipoprotein E-deficient (*ApoE*^−/−^) mice (atherosclerosis model mice) within 1 h, and obvious IR780 fluorescence could still be observed after 24 h, indicating the accumulation of the nanoassembly in the plaque area. In contrast, no obvious CFIR accumulation was observed at the carotid artery in healthy C57BL/6J mice. IR780 fluorescence and Oil red O (ORO) staining analysis of the isolated aorta further corroborated the enrichment of CFIR in the plaque region (Fig. 4d). Tissue section staining of the aortic arch and brachiocephalic artery clearly showed the nanoassembly (red) uptake by macrophages (blue) in the plaques (Fig. 4e), and such an uptake resulted in the accumulation of the CFIR nano-assembly in the atherosclerotic plaques. The targeted accumulation may be attributed to the enhanced endothelial permeability and active macrophages uptake in the plaque area.^50^ These results not only demonstrate the targeted enrichment ability of cargo-loading CF nanoassemblies in atherosclerotic plaque regions, but also suggest the wide application potential of the CF nanozyme due to its broad cargo-loading capacity. That is, when fluorescent probes are loaded, the obtained CF-based nanoassemblies may be used for the imaging analysis of diseases. If drugs are loaded, highly efficient treatment of diseases may be realized through the synergy of the drug and nanozyme.

Next, the metabolic pathway of cargo-loaded CF nanoassemblies was examined. *Ex vivo* experiments showed that the CFIR nanoassembly was enriched in macrophage-rich clearance organs, such as liver (Fig. S26†). To reveal the metabolic pathway of CF and CF-based nanoassemblies, we collected feces and urine samples of C57BL/6J mice at different time points after tail vein injection of CF and analyzed the Ce contents in these samples by ICP-MS. The results showed that a considerable amount of Ce was detected in the feces (Fig. S27†), suggesting that the nanoassemblies were mainly metabolized through the hepatobiliary system and excreted through the feces.^51^ Similar ICP-MS results were obtained from the feces and urine samples of CFP-injected mice, suggesting that CF and cargo-loaded CF nanoassemblies follow the same metabolic pathway.

### Anti-atherosclerotic efficacy *in vivo*

**Fig. 5:**
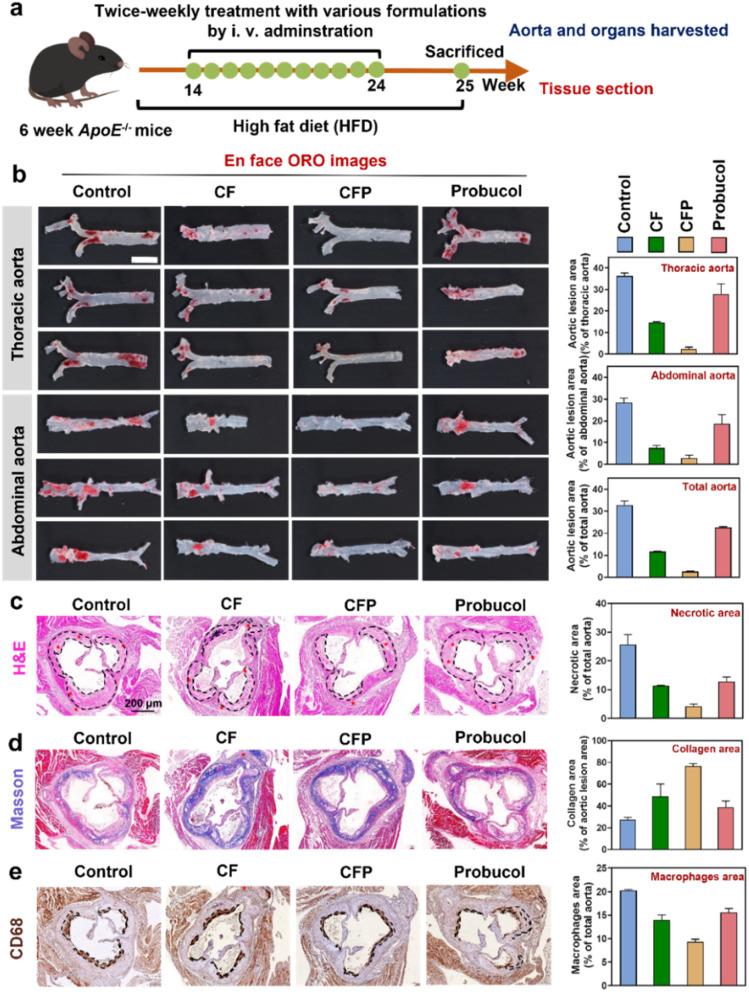
Anti-atherosclerotic efficacy *in vivo*. (a) Timeline of feeding and treatment of *ApoE*^−/−^ mice. (b) *Ex vivo* ORO-stained images of the aorta obtained from the *ApoE*^−/−^ mice treated with different formulations. The right images show the aortic lesion area as a percentage of the thoracic aorta, abdominal aorta and the total aorta. Scale bar: 1 cm. (c) Aorta root sections stained with H&E, (d) Masson and (e) CD 68. Scale bar: 200 µm. The right images show the necrotic area as a percentage of the total aorta area, collagen deposition area as a percentage of the aortic lesion area and macrophage infiltration area as a percentage of the total aorta.

As a moonlighting protein-like nanozyme, CF holds great promise for various biological and biomedical applications. Having demonstrated its application in imaging diagnosis of diseases by coassembling a fluorescent probe into the CF nanoassembly, we next aim to explore its feasibility for disease treatment by loading therapeutic drugs. In the above experiments, we have found that the probucol-loaded CFP nanoassembly exhibits superior ROS-eliminating ability and enhanced anti-inflammatory efficiency compared to the CF nanozyme and probucol. Based on the targeted accumulation behavior of CF-based nanoassembies in atherosclerotic plaques, CFP has shown great potential for the synergistic atherosclerosis treatment. To demonstrate this, the *ApoE*^−/−^ model mice were fed with a high-fat diet (HFD) for eight weeks to develop atherosclerotic plaques,^52^ and then divided into 4 groups. Each group received twice-weekly treatment with different formulations for 10 weeks (Fig. 5a). Compared to the control group without any treatment, in which the plaque area accounted for 35% of the total aorta area, probucol and CF nanozyme treatment could decrease the plaque area to 25% and 15%, respectively. It was exciting that CFP treatment was able to significantly minimize the plaque area to ∼9%. The markedly enhanced efficiency can be attributed to moonlighting protein-like properties of the nanoassembly (Fig. 5b). That is, its drug-loading and delivery capabilities enhance the enrichment of probucol at targeted sites, and the multiple enzyme activities in conjunction with the encapsulated probucol enable a synergistic treatment for atherosclerotic plaques. The sections of aortic roots in different groups were further analyzed to quantitatively assess the necrotic core, collagen deposition, and macrophage infiltration. As shown in Fig. 5c, CFP was more effective than the CF nanozyme and probucol in reducing the necrotic core area (marked by black dashed lines), showing the largest reduction degree from ∼25% to ∼4% of the total aortic area. Masson staining was used to observe the collagen deposition in the aortic lesion region. And again, the CFP-treated group showed the highest levels of collagen deposition, suggesting that CFP treatment may activate the repairing mechanism of the blood vessel wall, promoting the production of collagen and thus repairing damaged blood vessels (Fig. 5d). The increased collagen deposition can also stabilize the plaques, reducing the risk of plaque rupture and thrombosis. CD68 immunohistochemical staining can quantify macrophages in the plaque area, thus evaluating the development and deterioration of atherosclerosis (Fig. 5e). It was observed that treatment with CFP effectively reduced the macrophage infiltration area in the plaque region, preventing the pathological progression of atherosclerotic disease.^52^ Collectively, CFP can efficiently reduce the necrotic core area, promote the deposition of collagen, and inhibit macrophage infiltration, thus holding great promise for atherosclerosis management.

### Biosafety assessment

The biosafety of the CFP nanoassembly was investigated. As shown in Table S1,† there is no difference in the blood markers between CFP-treated and physiological saline-treated groups, and the expression levels of all these blood markers were within the normal range. Furthermore, the results of histological analysis showed that the histological features of the heart, liver, spleen, lung and kidney did not change (Fig. S26†). All these results indicated the excellent biosafety of the as-synthesized CFP nanoassembly.

## Conclusions

In summary, we developed a novel Ce-based nanozyme (referred to as CF) with high biostability through the assembly of Ce^3+^ with a phosphorylated amino acid derivative, followed by a straightforward heat treatment. The as-prepared CF nanozyme has both SOD and CAT activities, thus can work as a multifunctional nanozyme to catalyze cascade chemical reactions, and shows the abilities to eliminate ROS and modulate inflammation by inhibiting NF-κB pathway activation. Notably, in addition to its multiple enzymatic activities, CF can also serve as a versatile nanocarrier for the loading of various cargoes *via* a simple one-pot co-assembly reaction. The moonlighting protein-like multifunctionality makes cargo-loaded CF-based nanoassemblies hold great promise for a wide range of applications. As two examples, the near infrared fluorescent dye IR780-loaded CFIR nanoassembly was successfully employed for the imaging diagnosis of atherosclerotic plaques. The probucol-loaded CFP nanoassembly exhibited superior ROS-scavenging and anti-inflammatory effects compared to both the CF nanozyme and probucol, attributed to the synergy of the nanozyme and the drug, thus facilitating a highly efficient treatment of atherosclerosis. This work not only provides a novel Ce-based nanozyme with multifunctional properties akin to those of moonlighting proteins, but also establishes a new approach to endow nanozymes with additional functionalities, thereby expanding their potential applications.

## Data availability

The data that support the findings of this study are available in the ESI† of this article.

## Author contributions

G. H. conceived and designed the study. Q. C., D. K. and L. Z. supervised the research project. J. L. and W. J. performed the computational part. Z. D. provided essential resources for the western blot test. All authors participated in manuscript review and approval.

## Conflicts of interest

There are no conflicts to declare.

## Supplementary Material

SC-OLF-D5SC01014D-s001
