# Supplementary material for: Multifunctional cerium-based nanozymes as moonlighting protein mimics for atherosclerosis diagnosis and therapy
Source: Chem Sci. 2025 Apr 16;16(20):8772–82. doi: 10.1039/d5sc01014d (PMC12013363; doi:10.1039/d5sc01014d)
Supplement: SC-016-D5SC01014D-s001 [file SC-016-D5SC01014D-s001.pdf]

Supplementary Information (SI) for Chemical Science.
This journal is © The Royal Society of Chemistry 2025

Supporting information for

Multifunctional cerium-based nanozyme as moonlighting protein mimic for atherosclerosis diagnosis and therapy

Gui-Mei Han,[a,b] Jing-Qi Liu,[a] Zhi-Qi Dai,[a] Wei-Liang Jin,[a,c] Qi-Liang Cai,[d,*] De-Ming Kong,[a,*] Li-Na Zhu [c,*]

[a] *State Key Laboratory of Medicinal Chemical Biology, Tianjin Key Laboratory of Biosensing and Molecular Recognition, Research Center of Analytical Science, College of Chemistry, Nankai University, Tianjin, 300071, P. R. China.*

[b]*College of Chemistry and Chemical Engineering, Qilu Normal University, Jinan 250200, P. R. China.*

[c] *Department of Chemistry, School of Science, Tianjin University, Tianjin 300354, P. R. China.*

[d] *Department of Urology, The Second Hospital of Tianjin Medical University, Tianjin Institute of Urology, Tianjin 300211, P. R. China.*

Table of Contents

1. Experimental section

1.1 Chemicals and reagents

1.2 Construction of the **CF**

1.3 Molecular dynamics (MD) simulation

1.4 Construction of the **CFP**

1.5 Multiple enzymatic activities

1.6 Biocompatibility and intracellular uptake

1.7 ROS elimination

1.8 Inhibition of foam cell formation

1.9 Anti-inflammation by inhabiting the NF-κB pathway

1.10 Accumulation in the atherosclerotic plaques

1.11Anti-atherosclerotic efficacy in vivo

1.12 Biosafety assessment

2. Supplementary figures

Figure S1. Photos of the reaction systems containing different ratios of Ce^{3+} to Fmoc-O-phospho-L-tyrosine.

Figure S2. Chemical structure of Fmoc-O-phospho-L-tyrosine, Fmoc-L-tyrosine and O-phospho-L-tyrosine.

Figure S3. Tyndall phenomenon of the reaction solutions containing (i) Fmoc-O-phospho-L-tyrosine, (ii) Ce^{3+} and Fmoc-O-phospho-L-tyrosine, (iii) Ce^{3+} and Fmoc-L-tyrosine or (iv) Ce^{3+} and O-phospho-L-tyrosine.

Figure S4. Molecular dynamics (MD) simulation of the formation of **CF** nanoassembly.

Figure S5. ThT fluorescence of the **CF** nanoparticles (with or without heating treatment)

Figure S6. Stability of the **CF** nanoparticles with or without heating treatment in different solution systems.

Figure S7. Hydrodynamic diameters of **CF** nanoparticles after heating treatment for different time.

Figure S8. TEM images of **CF** nanoparticles with or without heating treatment.

Figure S9. XPS survey of **CF** nanoparticles with or without heating treatment.

Figure S10. XRD patterns of **CF** nanoparticles with or without heating treatment.

Figure S11. Elemental mapping images and energy dispersive spectrum of **CF** after heating treatment.

Figure S12. Radial distribution functions (RDF) for (a) the interaction between Ce^{3+} and carboxyl O, phosphate O, and carboxyl and phosphate O in **CF** with or without heating treatment.

Figure S13. Coordination number of Ce^{3+}-carboxyl in the **CF** nanoassemblies with or without heating treatment.

Figure S14. PMF values for the Ce^{3+}-carboxyl in the **CF** nanoassemblies with or without heating treatment.

Figure S15. High resolution Ce3d XPS spectra of **CF**.

Figure S16. HPLC chromatograms of probucol standard solution and **CFP.**

Figure S17. Stability and drug leakage of probucol from **CFP**

Figure S18. Drug release of probucol from **CFP**

Figure S19. Exacted mean fluorescence intensity (MFI) from the CLSM images in Figure 3.

Figure S20. The NF-*κ*B p-p65 to NF-*κ*B p65 ratios in the RAW 264.7 cells with different treatments.

Figure S21. Relative expression levels of iNOS, IL-1*β* and TNF-*α* proteins in the RAW 264.7 cells with different treatments (*β*-Actin as the control).

Figure S22. The percentage of ORO area in the RAW 264.7 cells with different treatments in Figure 3j.

Figure S22. Cytotoxicity of **CFP** towards RAW 264.7 and HUVEC cells.

Figure S23. Hemolysis test of **CFP.**

Figure S24. TEM image of CFIR.

Figure S25. Ex vivo images of different organs obtained from healthy C57BL/6J mice after 24 h post-injection of CFIR.

Figure S26. Contents of Ce in feces and urine detected by ICP-MS.

Table S2. Blood marker levels of healthy C57BL/6J mice after two weeks post-injection of

physiological saline, **CF**, **CFP** or probucol.

Figure S27. Histologic photograph of organs obtained from healthy C57BL/6J with different treatments.

3. Supplementary references

1. Experimental section

1.1 Chemicals and reagents

Ce $(NO_3)_3$, Fmoc-O-phospho-L-tyrosine, nitro-blue tetrazolium (NBT) and Riboflavin, ethylenediaminetetraacetic acid disodium (EDTA) were bought from Shanghai Macklin Biochemical Technology Co., Ltd. (Shanghai, China). Thioflavin T (ThT), Coumarin 6 (C6), Rhodamine B, IR780, Ce6, ICG and Probucol were purchased from MedChemExpress LLC. Lipopolysaccharides (LPS), 3%H_2O_2 and horseradish peroxidase (HRP) were obtained from Sigma-Aldrich (Shanghai, China). OxLDL weas purchased from Guangzhou Yiyuan Biotech. Co., Ltd. (Guangzhou, China). RIPA lysis buffer, SDS-PAGE sample loading buffer (5×), Hoechst 33342, cell count kit-8 (CCK-8) cell proliferation assay kit, JC-1 kit, Reactive Oxygen Species Assay Kit and BCA protein assay kit, were purchased from Beyotime Biotechnology Ltd. (Shanghai, China). LysoTracker™ Green was obtained from Thermo Fisher Scientific Inc. Dulbecco's Eagle's Medium (DMEM), fetal bovine serum (FBS) and 4% paraformaldehyde were obtained from Invitrogen Corp (Shanghai, China). All reagents were of at least analytical grade and used without additional purification unless specifically stated. All aqueous solutions were prepared with ultrapure water (Mill-Q, Millipore, 18.25 MΩ·cm).

1.2 Construction of CF

Stock solution of Ce^{3+} was prepared by dissolving Ce $(NO_3)_3{\cdot}6H_2O$ powder in water at a concentration of 20 mM. Fmoc-O-phospho-L-tyrosine was dissolved in DMF at a concentration of 100 mM. Typically, 20 μL of Fmoc-O-phospho-L-tyrosine solution and different volumes of Ce^{3+} were mixed in the tube and pure water was added to a volume of 1 mL, followed by adjusting pH to 7 by NaOH (1 M in water). Then the obtained nanoparticles were centrifugated at 8000 rpm for 10 min and redispersed in water for further use. For the heating treatment, the nanoparticles' solution was heating at 95 ^{o}C for different time on a thermo shaker incubator and then used for further analysis.

1.3 Molecular dynamics (MD) simulation

MD simulation was performed with the program GROMACS 2020.6.[1] The Lennard-Jones parameters are obtained by the OPLS-AA force field, and the water molecules are described by the three-point Optimal Point Charge (OPC3) water model.[2] The force-field parameters and geometry parameters of Fmoc-O-phospho-L-tyrosine were taken from the program sobtop 1.0(dev4) and described by the GAFF force field. We assumed that all Fmoc-O-phospho-L-tyrosine molecules in **CF** are partially deprotonated with two negative charges. The RESP atomic charge of Fmoc-O-phospho-L-tyrosine is calculated by Gaussian 16 at the level of B3LYP-D3(BJ)/6-311G* and combined with Multiwfn 3.8 (dev) program.[3] 40 Ce^{3+} and 60 Fmoc-O-phospho-L-tyrosine molecules are randomly inserted into the box of 50×50×50 $Å^3$. The simulation was started by minimizing the energies of the initial configuration using the steepest descent algorithm. After energy minimization, a 200 ps constant-NPT simulation (time step 0.5 fs) was performed to pre-equilibrate the system. Then, a 200 ns constant-NPT simulation (time step 2 ps) was carried out to obtain the equilibrium state at 298.15 K. Finally, a 110 ns constant-NPT simulation (time step 2 ps) was carried out to obtain the equilibrium state at 393.15 K. Periodic boundary conditions were employed for all x-y-z directions. The temperature control method uses Velocity-rescale hot bath to keep the temperature of the simulation.[4] The pressure was kept at 1 atm using a Parrinello-Rahman Barostat. The Particle Mesh Ewald (PME) summation technique was used to calculate the long-range electrostatic interactions.[5] The non-bonded interactions were calculated based on the Lennard-Jones (LJ) pair potentials, which assume interaction occurs between two bodies. LJ pair potentials were evaluated within a cut-off of 0.9 nm. The cross-interaction parameters were obtained from the Lorentz-Berthelot rules.[6] The simulated trajectories were visualized using VMD 1.9.3 software.[7]

1.4 Construction of CFP

Typically, 20 μL of Fmoc-O-phospho-L-tyrosine solution, 5 μL probucol solution (1 mg/mL) and 20 μL of Ce^{3+} (20 mM) were mixed in the tube and pure water was added to a volume of 1 mL, followed by adjusting pH to 7 by NaOH (1 M in water). Then, the obtained nanoparticles were centrifugated at 8000 rpm for 10 min and redispersed in water for further use. The obtained

nanoassembly was heated at 95 ^{o}C for 45 min.

The drug encapsulation efficiency (DEE) of probucol was detected by HPLC. The **CFP** assembly was dispersed in methanol to obtain free probucol molecules, and the DEE (%) was calculated as follows:

$$\mathrm{DEE}\ (\%) = \frac{\mathrm{PB\ in\ CFP}}{\mathrm{PB\ in\ feed}} \times 100\%$$

1.5 Multiple enzymatic activities

SOD activity. The SOD activity of the nanoassemblies was studied using nitrogen tetrazolium blue (NBT) photoreduction method. Riboflavin (10 μL, 12 mmol/L) was added to 15 μL of NBT (20 mmol/L), 400 μL of EDTA (0.1 mol/L) and 5.8 mL of pure water to obtain the detection solution. 50 μL of **CF** or **CFP** solution was mixed with 100 μL of detection solution. The mixture was shaken at 37 °C for 5 min and irradiated with a 40 W light for 2 min. Then, the absorbance at 600 nm was recorded.

CAT activity. The CAT activity of the nanoassemblies was studied using 10-acetyl-3,7-dihydroxyphenoxazine (Amplex™ Red) assay and the production of oxygen. Different concentrations of **CF** or **CFP** (measured by Ce, 10~50 μM) were mixed with H_2O_2 solution at a concentration of 100 mM. The final concentration of Amplex™ Red is 2.5 μg/mL. After reaction for 20 min, the fluorescence of the reaction solution was detected (λ_{ex} = 540 nm, λ_{em} = 590 nm). The fluorescence was negatively correlated with the concentration of residual H_2O_2 in the system.

$$\textbf{Amplex} + H_2O_2 \rightarrow \textbf{Resorufin (Fluorescent)}$$

Therefore, the oxygen content in the solution was measured using a portable dissolved oxygen meter. The O_2 concentration (unit: $mg \cdot L^{-1}$) in the solution was dynamically recorded in the following 20 min.

1.6 Biocompatibility and intracellular uptake

The biocompatibility of **CFP** was assessed using the CCK-8 cell viability kit assay. Briefly, HUVEC and RAW 264.7 cells were seeded in a 96-well plate at a density of 5000 cells per well,

and cultured for 12 h. The cells were then exposed to varied concentrations of **CFP** for 24 h. Subsequently, the CCK-8 solution was added to each well to a concentration of 10%, and the absorbance at 450 nm was measured. The well without **CFP** addition was defined as 100% and the well with DMEM only was defined as 0%.

To study the uptake of **CFIR** by macrophage cells. RAW 264.7 cells were seeded into confocal dishes and cultured overnight to facilitate adherence. After incubation with **CFIR** for different time, the cells were stained with Hoechst 33342 and a lysosome tracer, and subsequently analyzed using confocal microscopy.

1.7 ROS elimination

ROS elimination in RAW 264.7 cells. Simply, RAW 264.7 cells were seeded into CLSM dishes, incubated overnight, and treated by LPS for 12 h. Then, **CF, CFP or** probucol was added and incubated with the cells for 12 h. After washing the cells three times with PBS, 2,7-dichlorofluorescein diacetate (DCFH-DA, 10 μM) was added and incubated for 15 min. The cells were then washed with PBS again, and intracellular fluorescence was monitored using CLSM. The cells without any treatment were used as the control.

ROS elimination in HUVEC cells. HUVEC cells were seeded into CLSM dishes, incubated overnight, and treated by H_2O_2 for 8 h. Then, **CF, CFP or** probucol was added and incubated for 8 h. After washing the cells three times with PBS, DCFH-DA (10 μM) was added and incubated for 15 min. The cells were then washed with PBS again, and intracellular fluorescence was monitored using CLSM. The cells without any treatment were used as the control.

Analysis of mitochondria membrane potential (MMP). HUVEC cells were seeded into confocal dishes, incubated overnight at 37 °C, and then treated with H_2O_2 for 8 h. Then, **CF, CFP or** probucol was added and incubated for 8 h. After washing three times with PBS, the cells were incubated with JC-1 working solution for 20 min. After further washing with the buffer attached in the kit for several times, the cells were imaged by CLSM. The cells without any treatment waere used as the control.

1.8 Inhibition of foam cell formation

RAW 264.7 cells were seeded into CLSM dishes and incubated overnight. Then, the medium was replaced by a fresh medium containing oxLDL (100 μg/mL) and **CF, CFP** or probucol. After incubation for 48 h, the cells were washed three time with PBS, fixed with 4% paraformaldehyde for 20 min, and then stained with 0.3% ORO. The cells were washed with PBS three times again, and observed under an optical microscope. The cells without any treatment were used as control.

1.9 Anti-inflammation by inhabiting the NF-*κ*B pathway

Western blot (WB) assay, immunofluorescence imaging and RT-qPCR was selected to evaluate the inhibition of NF-*κ*B pathway by **CFP** and the anti-inflammatory capability of **CFP**.

Western blot (WB) assay. Western blot (WB) assay was used to evaluate the phosphorylation of NF-*κ*B p65 and the levels of inflammatory factors. RAW 264.7 cells were seeded into a 24-well plate and incubated overnight for cell adherence. Then, the cells were stimulated with 10 μg/mL LPS and further incubated with **CF**, **CFP** or probucol for 12 h. The cells without any treatment were used as control. Then, the cells were collected. Intracellular proteins were extracted using RIPA lysis buffer on ice, and the protein concentration was determined using a bicinchoninic acid (BSA) protein detection kit. The proteins were separated by sodium dodecyl sulfate-polyacrylamide gel electrophoresis (SDS-PAGE) and transferred to a polyvinylidene fluoride membrane. After blocking with 5% milk in PBST for 2 h, the membrane was incubated overnight with an NF-*κ*B p65, NF-*κ*B phospho-NF-*κ*B p65 antibody, iNOS antibody, TNF-*α* antibody, IL1*β* antibody, and *β*-Actin antibody on a shaker at 4 °C and then incubated for 2 h at room temperature with corresponding secondary antibodies. After three washes with PBST and an incubation with enhanced chemiluminescence reagent, the membrane was visualized using a chemiluminescent imaging system.

Immunofluorescence staining. Immunofluorescence imaging was performed for NF-*κ*B p65 translocation to evaluate inhibition of NF-*κ*B pathway. The cells were stimulated with 10 μg/mL LPS and further incubated with **CF**, **CFP** or probucol for 12 h. The cells without any treatment were used as control. Then, the cells were washed three times with PBS, followed by fixing with 4% paraformaldehyde for another 30 min. After that, the cells were treated by a membrane

breaking solution containing 1% Triton. After a 30 min treatment, the cells were washed three times with PBS, followed by incubation with diluted primary antibody in dark for 12 h. Subsequently, the cells were washed three times with PBS and then incubated with diluted Alexa Fluor® 488-conjugated secondary antibody solution in dark for another 1 h. Finally, after washing three times with PBS, the cells were stained with Hoechst 33342, and then imaged using a fluorescence microscope.

RNA extraction and quantitative PCR analysis. Quantitative PCR was used to measure specific gene expression during inflammatory process.[8] RAW 264.7 cells were seeded on 6-well plate and incubated overnight for cell adherence. The cells were stimulated with 10 μg/mL LPS and further incubated with **CF**, **CFP** or probucol for 12 h. The cells were then washed three times with PBS and the total RNA was extracted using a Trizol kit. The levels of inflammatory mRNAs were determined by RT-PCR. The data were analyzed by the $2^{-\Delta\Delta CT}$ method, and the values were normalized to the mean expression of *β*-Actin. The primer sequences of specific inflammatory-related genes were listed in Table S1.

Table S1. Primer sequences used for real-time PCR in this study[8]

Gene	Forward Sequencing Primer (5' to 3')	Reverse Sequencing Primer (5' to 3')
iNOS	CAGCTGGGCTGTACAAACCTT	CATTGGAAGTGAAGCGTTTCG
IL-1*β*	TGCCACCTTTTGACAGTGATG	AAGGTCCACGGGAAAGACAC
TNF-*α*	GGACTAGCCAGGAGGGAGAA	CGCGGATCATGCTTTCTGTG
β-Actin	AGCCCATCCTTCGAGTACAAA	TCTTGGTGCGATAACTGGTGG

1.10 Accumulation in atherosclerotic plaques

All the animal experiments were performed under the Care and Use of Laboratory Animals guidelines by the Institute of Radiation Medicine Chinese Academy of Medical Sciences. The accreditation number of the laboratory is SYXK(Jin) 2019-0002. Apolipoprotein E-deficient ($ApoE^{-/-}$) mice and healthy C57BL/6J mice (six weeks old, male) were purchased from Sibeifu biotechnology Co. Ltd (Beijing, China). To induce atherosclerotic plaque formation, the $ApoE^{-/-}$

mice were fed a high-fat diet (HFD, 1.25% cholesterol, 20% fat, 45% carbohydrate, and 23% protein, Research Diets Inc, D12108C) for 11-12 weeks.

After the injection of IR780-loaded **CFIR** (200 μL, 10 mM), the mice were anesthetized and settled at supine and prone position. *In vivo* NIR-II images were obtained at different time points. Then, the aortas and organs were obtained after 24 h post-injection. Then NIR-II images and Oil Red O (ORO) stained images were obtained. The 6-μm-thick frozen tissue sections of brachiocephalic arteries and aortic arches were also observed under the Nikon $A1^+$ to locate the **CFIR** nanoparticles.

After the injection of **CFIR** (200 μL, 10 mM), the mice were housed in metabolic cages and the feces and urines were collected at different time points. Then, the dried feces samples and urine were digested in 5% HNO_3, followed by ICP-MS test.

1.11 Anti-atherosclerotic efficacy *in vivo*

After 8 weeks, *ApoE*$^{-/-}$ mice was divided into 4 groups (3 in each group) and all mice were kept a HFD diet. Three groups of mice were subjected to a twice-weekly injection of different formulations (**CF**, **CFP** or probucol).

After 11 weeks, all the aortas were harvested. The aortas were dissected and stained with ORO for 30 min. Then, the images were obtained using optical microscope. The serial tissue sections of the aortic roots were fixed with paraformaldehyde for 30 minutes, embedded in OCT, frozen at -20 °C for 2 hours, sliced at a thickness of 5 μm, and then separately stained with hematoxylin-eosin (H&E), Masson's trichrome and antibodies to CD68 for histology and immunohistochemistry evaluation.

1.12 Biosafety assessment

For histopathological study, major organs (heart, liver, spleen, lung and kidney) of C57BL/6 mice were harvested at day 14 post injection of different formulations (**CF**, **CFP** or probucol), and then performed H&E staining analysis to observe the pathological damage. Routine blood tests are performed by collecting whole blood at day 14 post injection of different formulations (**CF**, **CFP** or probucol). PBS buffer-treated group was used as a control.

2. Supplementary figures

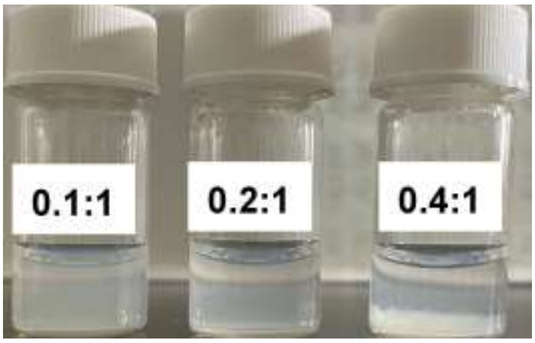

Figure S1. Photos of the reaction systems containing different ratios of Ce^{3+} to Fmoc-O-phospho-L-tyrosine.

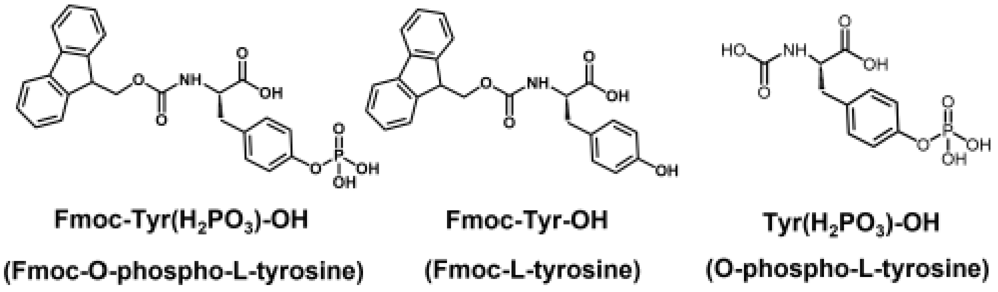

Figure S2. Chemical structure of Fmoc-O-phospho-L-tyrosine, Fmoc-L-tyrosine and O-phospho-L-tyrosine.

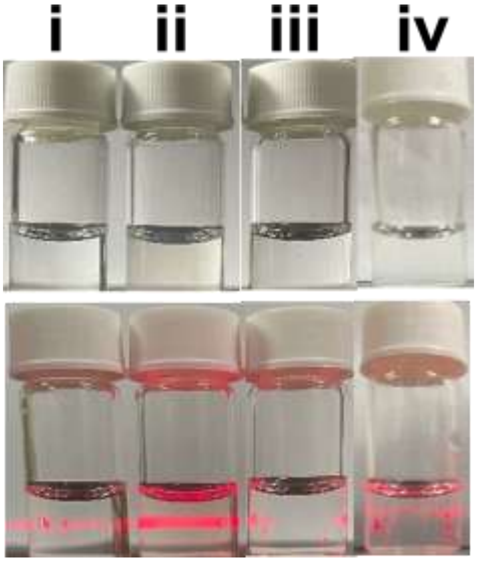

Figure S3. Tyndall phenomenon of the reaction solutions containing (i) Fmoc-O-phospho-L-tyrosine, (ii) Ce^{3+} and Fmoc-O-phospho-L-tyrosine, (iii) Ce^{3+} and Fmoc-L-tyrosine or (iv) Ce^{3+} and O-phospho-L-tyrosine.

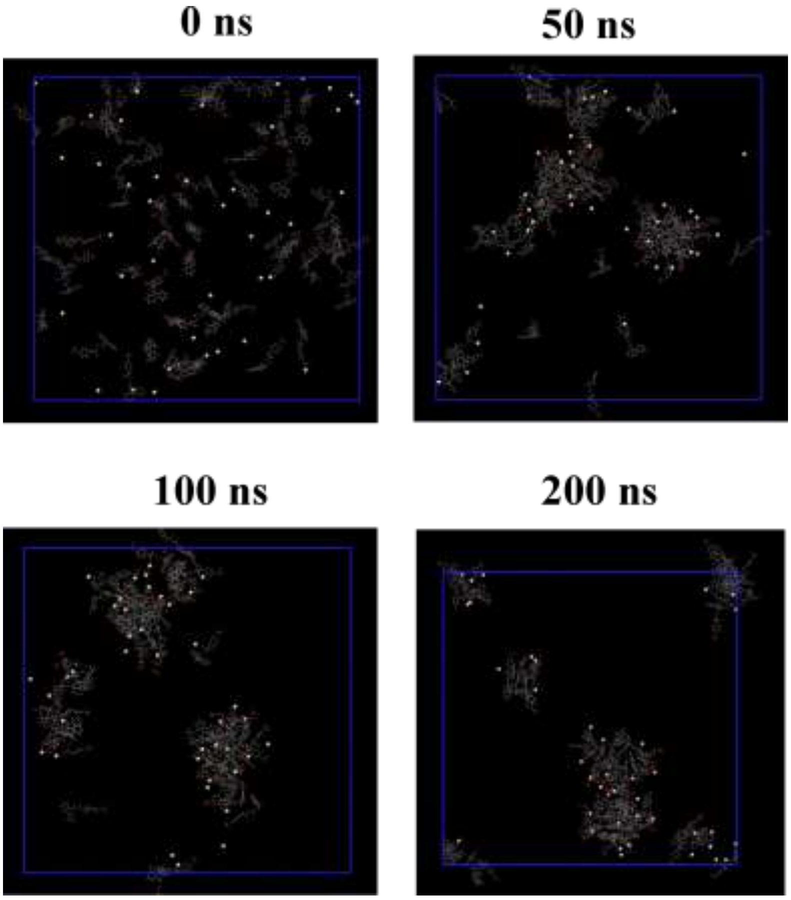

Figure S4. Molecular dynamics (MD) simulation of the formation of **CF** nanoassembly.

Free thioflavin T (ThT) almost emits no fluorescence. When ThT is co-assembled into CF nanoparticles through π-π stacking with the Fmoc aromatic structure, its molecular vibration is inhibited, thus greatly enhancing the fluorescence.[9] The disintegration of **CF** nanoparticles will result in the release of ThT molecules, leading to the fluorescence decrease of the **CF** solution.

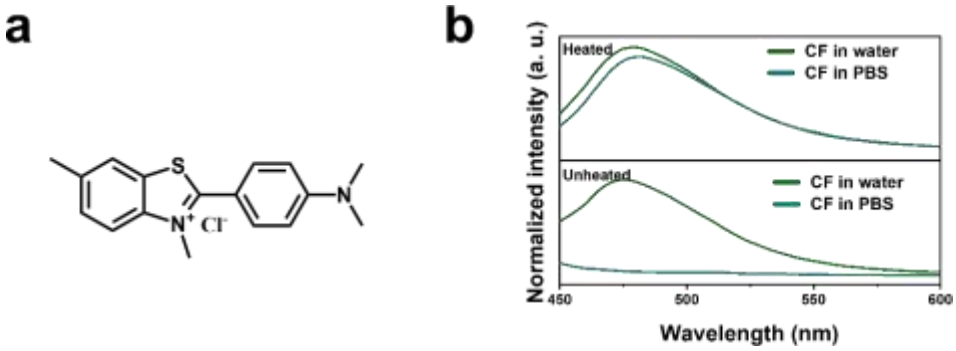

Figure S5. (a) Chemical structure of ThT. (b) ThT fluorescence of the **CF** nanoparticles (with or without heating treatment) after incubation in water or PBS buffer at 37 °C for 6 h.

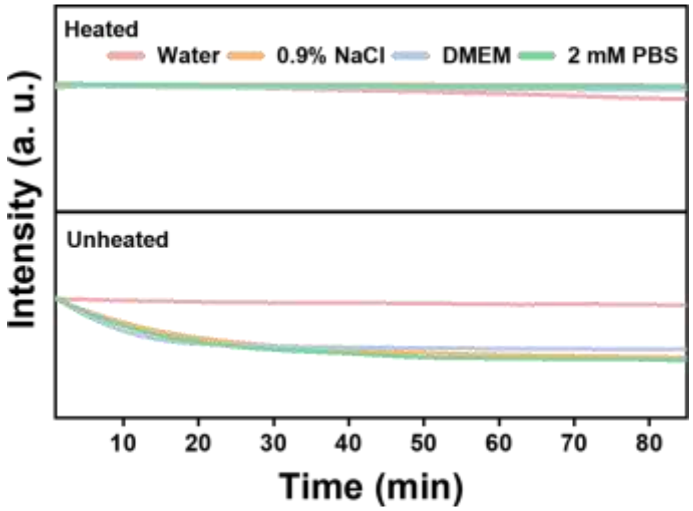

Figure S6. Stability of the **CF** nanoparticles with or without heating treatment in different solution systems.

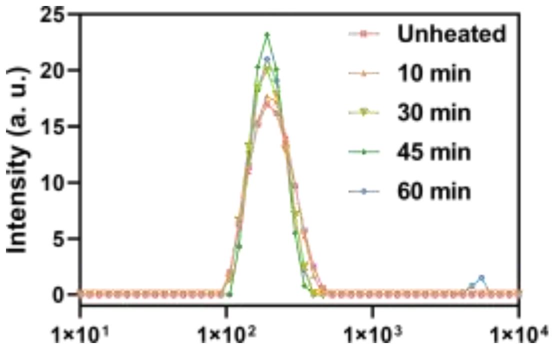

Figure S7. Hydrodynamic diameters of **CF** nanoparticles after heating treatment for different time.

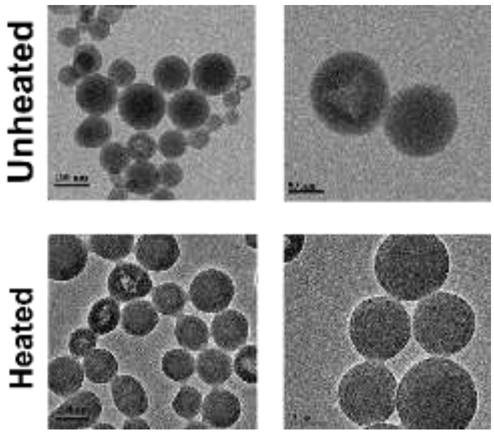

Figure S8. TEM images of **CF** nanoparticles with or without heating treatment.

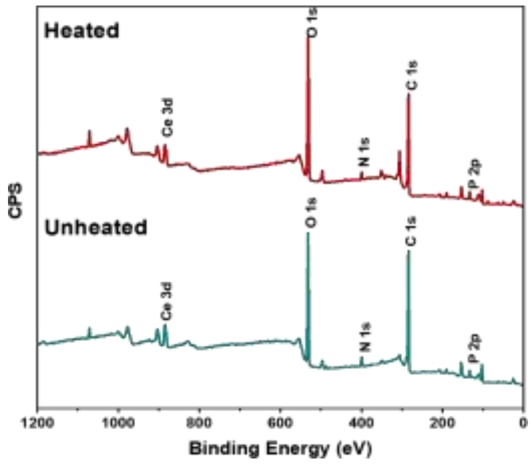

Figure S9. XPS survey of **CF** nanoparticles with or without heating treatment.

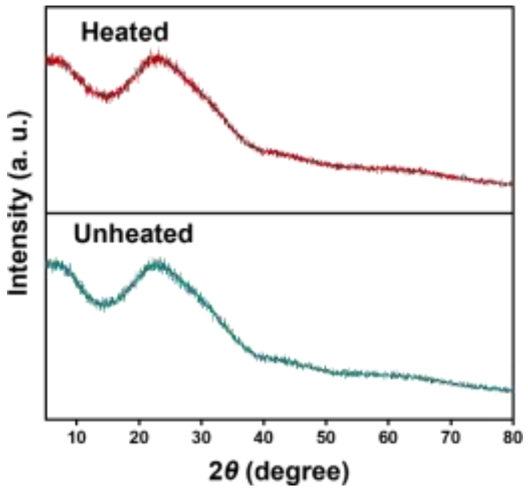

Figure S10. XRD patterns of **CF** nanoparticles with or without heating treatment.

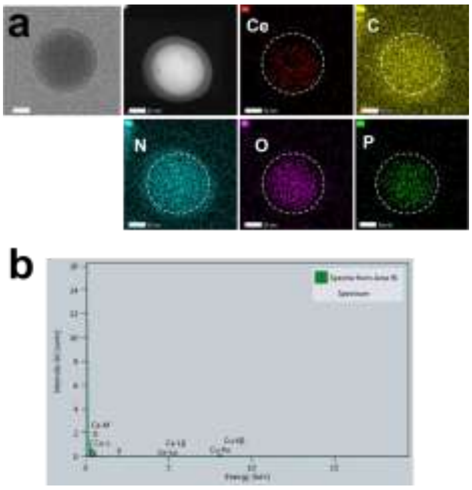

Figure S11. (a) Elemental mapping images and (b) energy dispersive spectrum of **CF** after heating treatment. Scale bar, 50 nm.

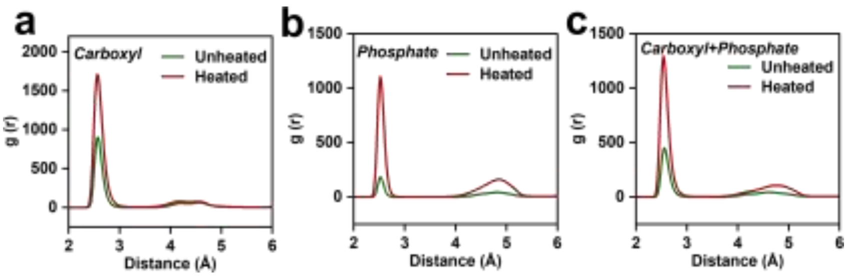

Figure S12. Radial distribution functions (RDF) for (a) the interaction between Ce^{3+} and carboxyl O, (b) the interaction between Ce^{3+} and phosphate O, and (c) the combined interactions of Ce^{3+} with carboxyl and phosphate O in **CF** with or without heating treatment.

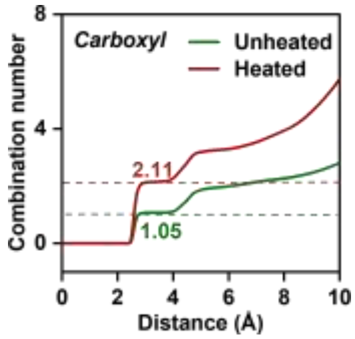

Figure S13. Coordination number of Ce^{3+}-carboxyl in the **CF** nanoassemblies with or without heating treatment.

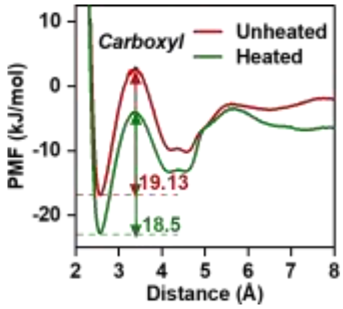

Figure S14. PMF values for the Ce^{3+}-carboxyl in the **CF** nanoassemblies with or without heating treatment.

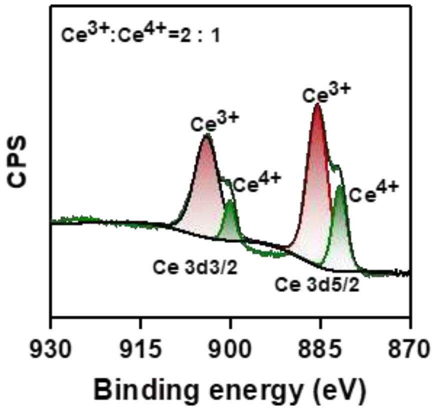

Figure S15. High resolution Ce3d XPS spectra of **CF**.

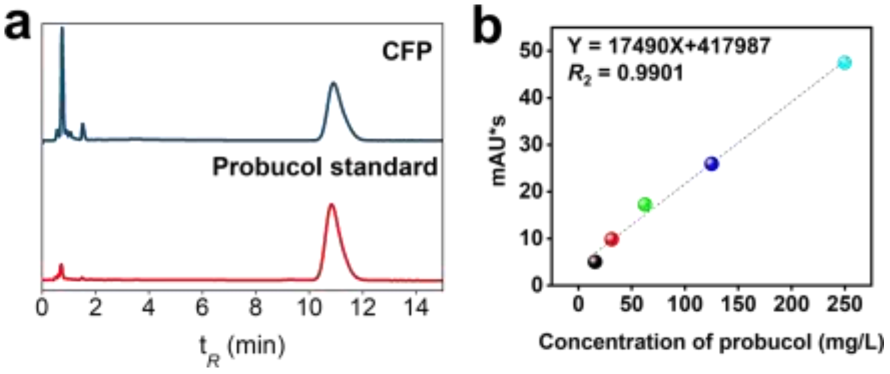

Figure S16. (a) HPLC chromatograms of probucol standard solution and **CFP.** (b) Standard curve of probucol.

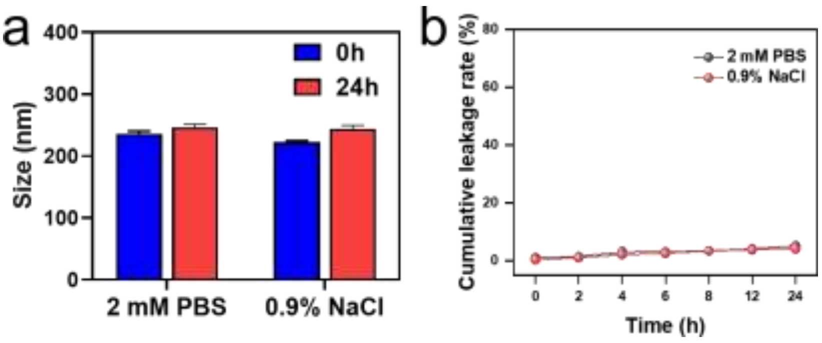

Figure S17. (a) Hydrodynamic diameter of **CFP** before and after incubation in 2 mM PBS and 0.9% NaCl for 24 hours. (b) Leakage rate of probucol from **CFP** during 24 hours of incubation.

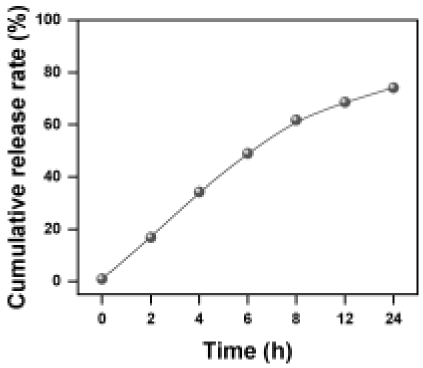

Figure S18. Release profile of probucol from **CFP** in 10 mM PBS

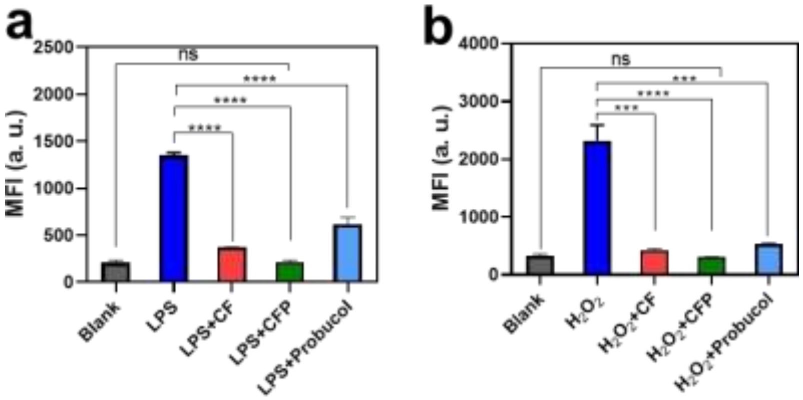

Figure S19. (a) Exacted mean fluorescence intensity (MFI) from the CLSM images in Figure 3a. (b) Figure 3b.

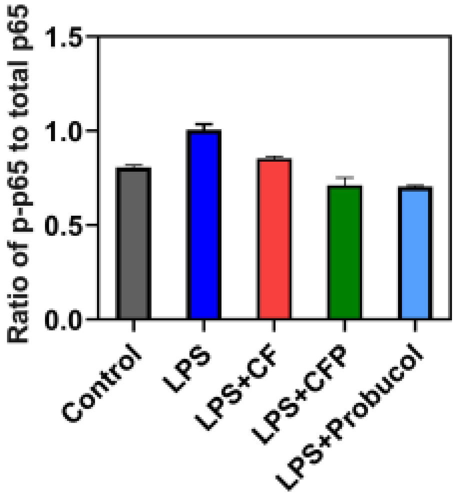

Figure S20. The NF-κB p-p65 to NF-κB p65 ratios in the RAW 264.7 cells with different treatments.

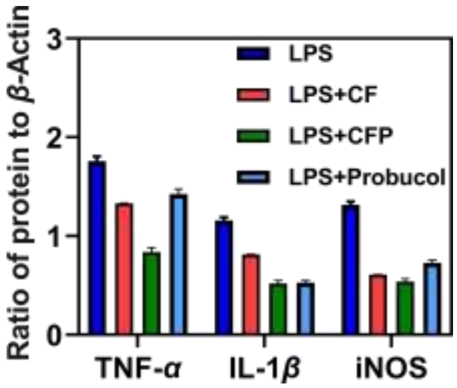

Figure S21. Relative expression levels of iNOS, IL-1β and TNF-α proteins in the RAW 264.7 cells with different treatments (β-Actin as the control).

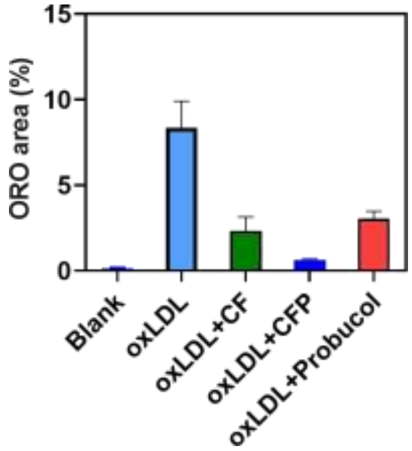

Figure S22. The percentage of ORO area in the RAW 264.7 cells with different treatments. Corresponding cell images are shown in Figure 3j.

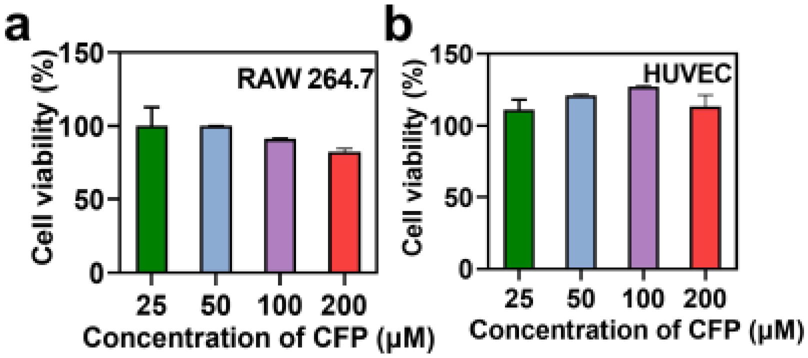

Figure S23. Cytotoxicity of **CFP** towards (a) RAW 264.7 and (b) HUVEC cells.

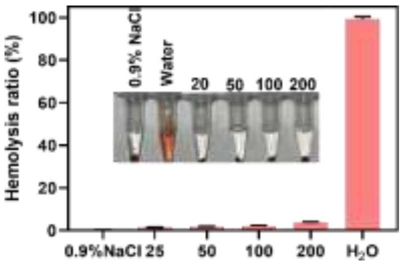

Figure S24. Hemolysis test of **CFP.**

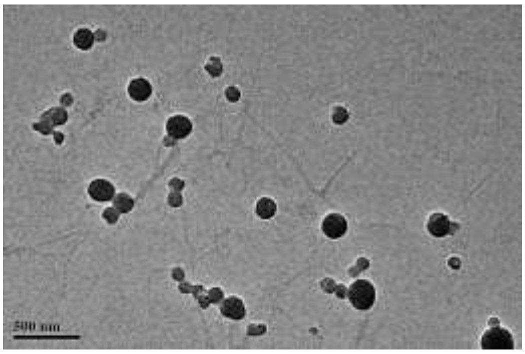

Figure S25. TEM image of **CFIR**. scale bar: 500 nm.

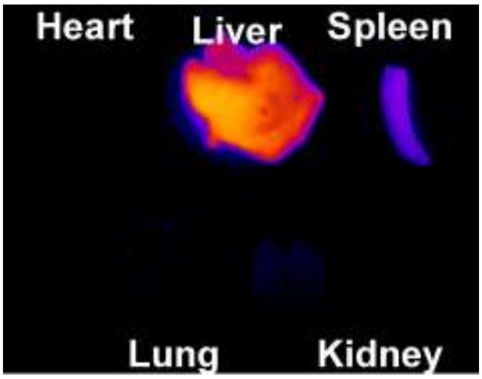

Figure S26. *Ex vivo* images of different organs obtained from healthy C57BL/6J mice after 24 h post-injection of **CFIR**.

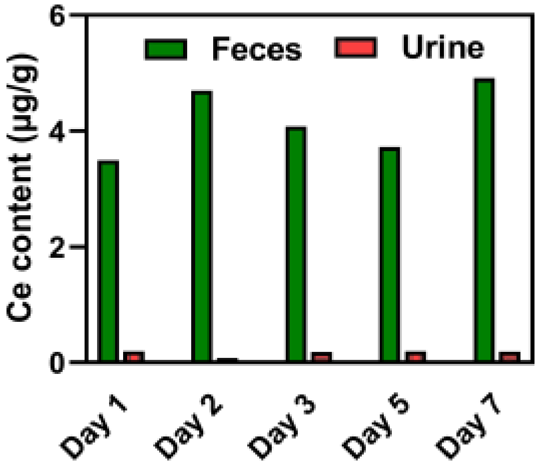

Figure S27. Contents of Ce in feces and urine detected by ICP-MS.

Table S2. Blood marker levels of healthy C57BL/6J mice after two weeks post-injection of physiological saline, **CF**, **CFP** or probucol

Group	PLT (10^{11}/L)	WBC (10^{9}/L)	MCHC (10 g/L)	MCH (pg)	MCV (fL)
Reference	4~16	0.8~10.6	30~36	13~18	41~55
Control	12.81	2.79	35.4	16.2	45.7
CF	7.01	2.31	34.5	15.9	46
CFP	6.77	2.97	36.3	16.7	45.9
Probucol	10.55	3.72	33.9	15.4	45.2

Reference represents the given normal range of different markers. Platelet (**PLT**), white blood cell (**WBC**), mean corpuscular hemoglobin concentration (**MCHC**), mean corpuscular hemoglobin (**MCH**), mean corpuscular volume (**MCV**).

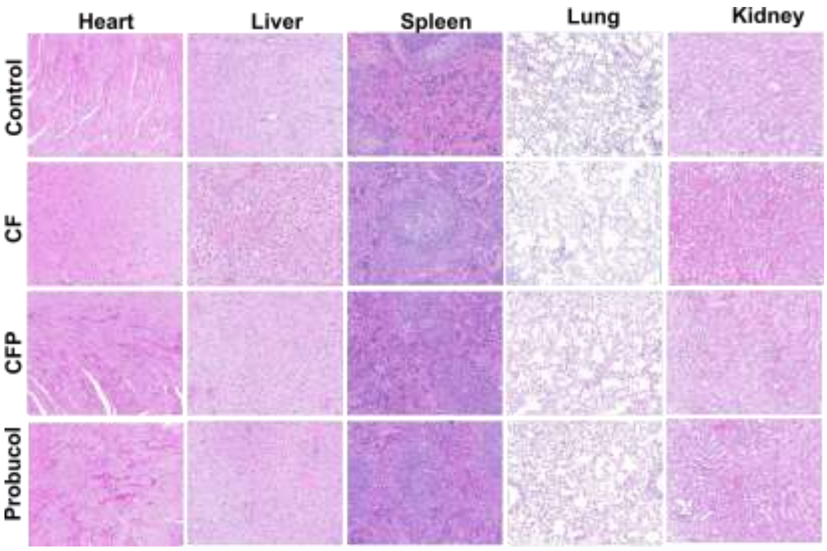

Figure S28. Histologic photograph of organs obtained from healthy C57BL/6J with different treatments.

3. Supplementary references

1. Abraham, M. J.; Murtola, T.; Schulz, R.; Páll, S.; Smith, J. C.; Hess, B.; Lindahl, E., GROMACS: High performance molecular simulations through multi-level parallelism from laptops to supercomputers. *Software X*, 2015, ***1-2***, 19-25.

2. Izadi, S.; Anandakrishnan, R.; Onufriev, A. V., Building water models: a different approach. *J. Phys. Chem. Lett.*, 2014, ***5*** (21), 3863-3871.

3. Lu, T.; Chen, F., Multiwfn: a multifunctional wavefunction analyzer. *J. Comput. Chem.*, 2012, ***33*** (5), 580-92.

4. Fly, A.; Kirkpatrick, I.; Chen, R., Low temperature performance evaluation of electrochemical energy storage technologies. *Appl. Therm. Eng.*, 2021, ***189***, 116750.

5. Cisneros, G. A.; Karttunen, M.; Ren, P.; Sagui, C., Classical electrostatics for biomolecular simulations. *Chem. Rev.*, 2014, ***114*** (1), 779-814.

6. Forsman, J.; Woodward, C. E., Limitations of the derjaguin approximation and the Lorentz-Berthelot mixing rule. *Langmuir*, 2010***, 26*** (7), 4555-4558.

7. Wang, Y.; Kiziltas, A.; Blanchard, P.; Walsh, T. R., Calculation of 1D and 2D densities in VMD: A flexible and easy-to-use code. *Comput. Phys. Commun.*, 2021***, 266***, 108032.

8. Yang, B.; Yao, H.; Yang, J.; Chen, C.; Guo, Y.; Fu, H.; Shi, J., In situ synthesis of natural antioxidase mimics for catalytic anti-inflammatory treatments: rheumatoid arthritis as an example. *J. Am. Chem. Soc.*, 2022, ***144*** (1), 314-330.

9. Arad, E.; Green, H.; Jelinek, R.; Rapaport, H., Revisiting thioflavin T (ThT) fluorescence as a marker of protein fibrillation - The prominent role of electrostatic interactions. *J Colloid Interface Sci.*, 2020, ***573***, 87-95.
